# Heat health assessment and risk simulation prediction in eastern China: a geospatial analysis

**DOI:** 10.3389/fpubh.2025.1521997

**Published:** 2025-03-07

**Authors:** Ming Li, Jiaying Xu

**Affiliations:** ^1^School of Public Health, Jilin University, Changchun, China; ^2^The Second Hospital of Dalian Medical University, Dalian, China

**Keywords:** heat health risk, CA-Markov model, social vulnerability, urban heat, spatial-temporal changes

## Abstract

**Background:**

High temperatures pose significant health risks and societal challenges in China, with spatial variations in heat health risks. Furthermore, due to the constraint imposed by heat health risk assessment on the construction of the public health security framework, it is necessary to explore the heat health risk pattern of spatial distribution and the trend of future risk development in eastern China.

**Methods:**

Based on the Intergovernmental Panel on Climate Change (IPCC) and Risk Triangle framework which is combined with natural and socio-economic factors, the heat health risk assessment index system of eastern China is established in this paper. This paper enhances the accuracy of risk maps with the aid of high-resolution imagery. It also focuses specifically on the exposure of construction workers in urban areas and agricultural workers in rural areas. This paper also evaluates the heat health risk of eastern China from 2010 to 2019 by using ArcGIS and the CA-Markov model.

**Results:**

The heat health risk in most areas of eastern China is predominantly highest risk, with the proportion of highest and medium risk areas increasing steadily from 2010 to 2019. The spatial distribution pattern reveals that high-risk areas are concentrated in the central urban areas, while low-risk areas are primarily in the mountainous regions, suburbs, rural areas, and water source areas. The conversion of heat health risk areas mainly occurs between adjacent levels, with no mutation process. From 2010 to 2025, the heat health risk of eastern China has been improving, and the overall distribution pattern of risk levels remains consistent.

**Conclusion:**

The research findings provide a basis for us to gain a deeper understanding of the vulnerability of different groups. This study not only presents spatial distribution maps of health risks, but offers a new perspective for us to comprehend the complexity and diversity of these risks. The research findings also establish a foundation for optimizing monitoring and warning systems. Furthermore, this study provides scientific evidence for policymakers to develop comprehensive heatwave mitigation plans. Nevertheless, we must acknowledge the limitations of the research and recognize that there is room for improvement in the future.

## 1 Background

Since 1980s, the global area affected by heatwaves has tripled compared to that in previous periods (1). A nationwide analysis in the United States has shown that during heat waves, mortality increases by 3.74%, with this effect being even more pronounced in specific regions such as the Northeast and Midwest (2). Greenhouse gas emissions have intensified heatwaves in Europe and North America, and these events are projected to worsen in the future (3). Moreover, the characteristics of heat waves—including their intensity, duration, and timing within the season—significantly influence their impact on health. For example, a 1°F increase in heat wave intensity has been linked to a 2.49% increase in mortality risk, while each additional day of a heat wave contributes to a 0.38% increase in risk (4). In one study, considering the impact of a single heat wave, it was estimated that during three heat waves in 2013, a total of 679 extra heat-related illnesses occurred, relative to the average values for the corresponding periods in previous years (5). However, the challenges posed by heat waves are not static. Over the past few decades, many regions, particularly in China, have experienced longer, stronger, and more frequent heat waves. Northern China has witnessed dramatic increases in intensity, while southern regions have seen significant lengthening in duration (6). These trends are alarming and point to a future where heat waves may become even more devastating to human health. Additionally, urban areas experience heatwave worsening due to the urban heat island (UHI) effect. The UHI effect not only raises temperatures but also exacerbates the health impacts of extreme heat conditions, leading to higher mortality rates in urban areas (7). The heat island effect during a heatwave not only raises ambient temperatures but also amplifies the disparity between urban and rural temperatures (8), with UHI being more pronounced at night (9). Urban centers with high population densities exposed to the UHI effect face substantial health risks (10).

In response to the increasingly frequent extreme heat events, heat health risk assessment has emerged as a research hotspot. Current research has developed a comprehensive assessment framework (11–14) that encompasses heat hazards, social vulnerability, and population exposure (15–17). In past research, detailed heat health risk assessments at the city level in the Philippines were conducted. By integrating remote sensing data with socio-ecological indicators, the aim was to provide information to policymakers and urban planners about the most vulnerable cities and to guide them in prioritizing adaptation measures (18). Hu et al. (19) overcame the limitations of traditional heat health risk assessments by using fine spatial scale data to improve the accuracy and spatial resolution of risk maps. Researchers have carried out a comprehensive spatial evaluation of heat health risks in Hong Kong, utilizing Principal Component Analysis (PCA) to categorize various indicators into meaningful groups. Through this process, they have developed a highly refined Heat-Related Health Risk Index (HHRI) (20). Other researchers have conducted a systematic review and meta-analysis of epidemiological evidence, focusing specifically on the impact of increases and decreases in ambient temperature on mortality and morbidity rates among individuals aged 65 and above. Their objective was to quantify the risks associated with temperature changes across various health outcomes (21). Although studies have begun to focus on vulnerable groups (22–25), research that comprehensively considers all exposed populations and develops mitigation measures based on their unique vulnerabilities is still inadequate (26, 27). This has led to past research being often constrained by region- and population-specific factors when providing detailed recommendations. The accuracy of population data is crucial for the effectiveness of assessments (28), but current research is often limited by census data (29, 30) and issues of uneven population distribution (31). Additionally, existing research primarily focuses on current and past risks (32–34), lacking spatial prediction methods for identifying future heat risk areas. These methods are crucial for adaptive planning and risk management at the spatial grid level, but they have not yet been fully developed (18, 35). The Sixth Assessment Report (AR6) of the Intergovernmental Panel on Climate Change (IPCC) provides a comprehensive update on climate change science (36), while the Risk Triangle framework sheds light on various factors contributing to risky behaviors, such as hazards, vulnerability, and exposure (37). These factors can be more broadly applied to understand and address risks associated with climate change. Therefore, incorporating spatial prediction methods into heat health risk assessments is expected to improve the effectiveness and practicality of the assessments.

## 2 Study aim

To address the aforementioned research gaps, our objective is to systematically assess the heat health risks in eastern China and make predictions about future heat health risks. This study covers a wide range of data from 2010, 2015, and 2019, encompassing socio-economic, remote sensing, health resources, vulnerable groups, and natural resources. In terms of methodology, this study employed PCA to determine the weight of each indicator. Additionally, the CA-Markov model was utilized for simulating and predicting heat health risks. Exploring health risk zoning will provide a basis for policy-making and promote sustainable development.

## 3 Material and methods

### 3.1 Study area

**Figure 1:**
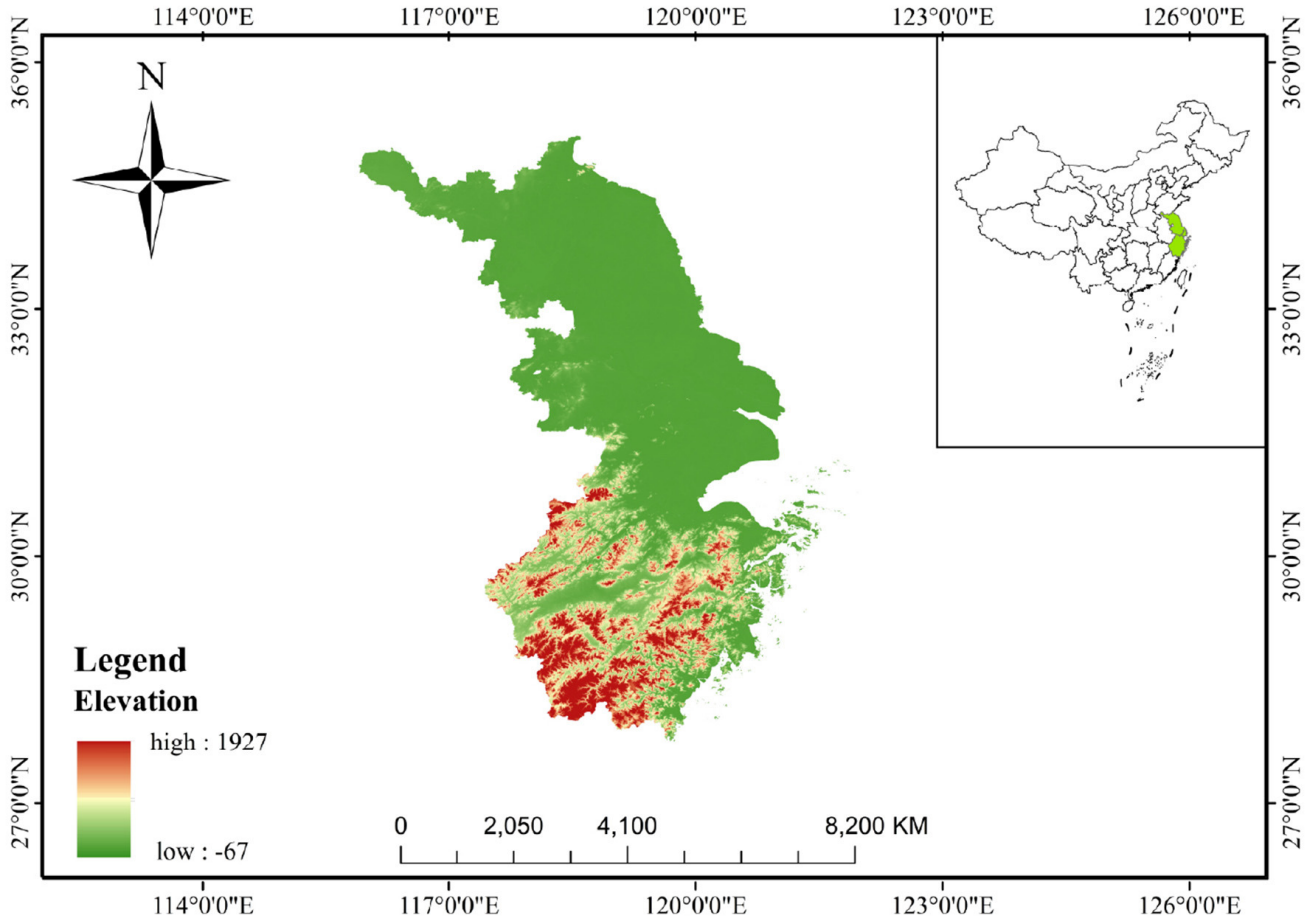
Geographical location of the eastern China.

Eastern China (Figure 1), covering Jiangsu, Shanghai, and Zhejiang, is located at longitudes 116°18′-123°10′E and latitudes 27°02′-35°20′N. It has a total area of ~219,000 km^2^ and resident population of ~170 million. Eastern China is characterized by several factors that collectively increased heat health risk. Firstly, it has a highly developed economy with rapid development. Secondly, it has an extremely high population density, accompanied by a concentrated population distribution. Thirdly, it shows a high level of urbanization, which leads to a significant urban heat island effect. Lastly, its coastal location makes it susceptible to the influence of the marine climate.

### 3.2 Data source

In order to explore the heat health risk pattern of eastern China in recent years, considering the availability of data and referring to previous related studies, this paper selected 2010–2019 as the research period, and 2010, 2015 and 2019 were selected as the time nodes to collect and analyze the data of these three periods and reflect the heat health risk pattern of eastern China in the last years. The data used in this study (Table 1) cover the socio-economic, remote sensing, health resources, vulnerable groups and natural resources data in 2010, 2015 and 2019. The normalized difference vegetation index (NDVI), land surface temperature (LST), gross national product (GDP) and population density data are derived from the Resource and Environmental Science Data Center of the Chinese Academy of Sciences. The elevation data is from the geospatial data cloud (GSclould) and is 90 m resolution digital elevation product data. The disposable income, physicians, hospital beds, older adults and female populations, water resources, unemployment rate and agricultural and construction practitioner data are obtained from the statistical yearbook of eastern China and in ArcGIS10.7, with a resolution of 1 km.

**Table 1:** Heat health risk assessment indicators.

**Factor category**	**Indicator**	**Unit**	**Source**	**Attribute**	**Spatial resolution**
Heat hazard	LST	°C	http://www.resdc.cn/	+	1KM
Social vulnerability	GDP (SV1)	Yuan	http://www.resdc.cn/	–	1KM
	Disposable income (SV2)	Yuan	Zhejiang Statistical Yearbook Jiangsu Statistical Yearbook Shanghai Statistical Yearbook(SV2–7)	–	1KM
	Physicians (SV3)	Man		–	1KM
	Hospital beds (SV4)	Individual		–	1KM
	Older adults (SV5)	%		+	1KM
	Female (SV6)	%		+	1KM
	Unemployment (SV7)	%		+	1KM
Human exposure	NDVI (EX1)	/	http://www.resdc.cn/(EX1–2)	–	1KM
	Population density (EX2)	Man/m^2^		+	1KM
	Water resources (EX3)	m^3^	Zhejiang Statistical Yearbook Jiangsu Statistical Yearbook Shanghai Statistical Yearbook(EX3–5)	–	1KM
	Agricultural practitioners (EX4)	%		+	1KM
	Construction practitioners (EX5)	%		+	1KM

“+” means positive action; “–” means reverse action.

The original data are projected into the same coordinate system (WGS_1984) through ArcGIS10.7 and are unified into the same spatial boundary that equals the boundary of the research area by cutting. The above process of data preprocessing was carried out in ArcGIS 10.7. Finally, the data are resampled to the same spatial resolution of 1 km × 1 km by means of nearest neighbor.

### 3.3 Assessment framework

**Figure 2:**
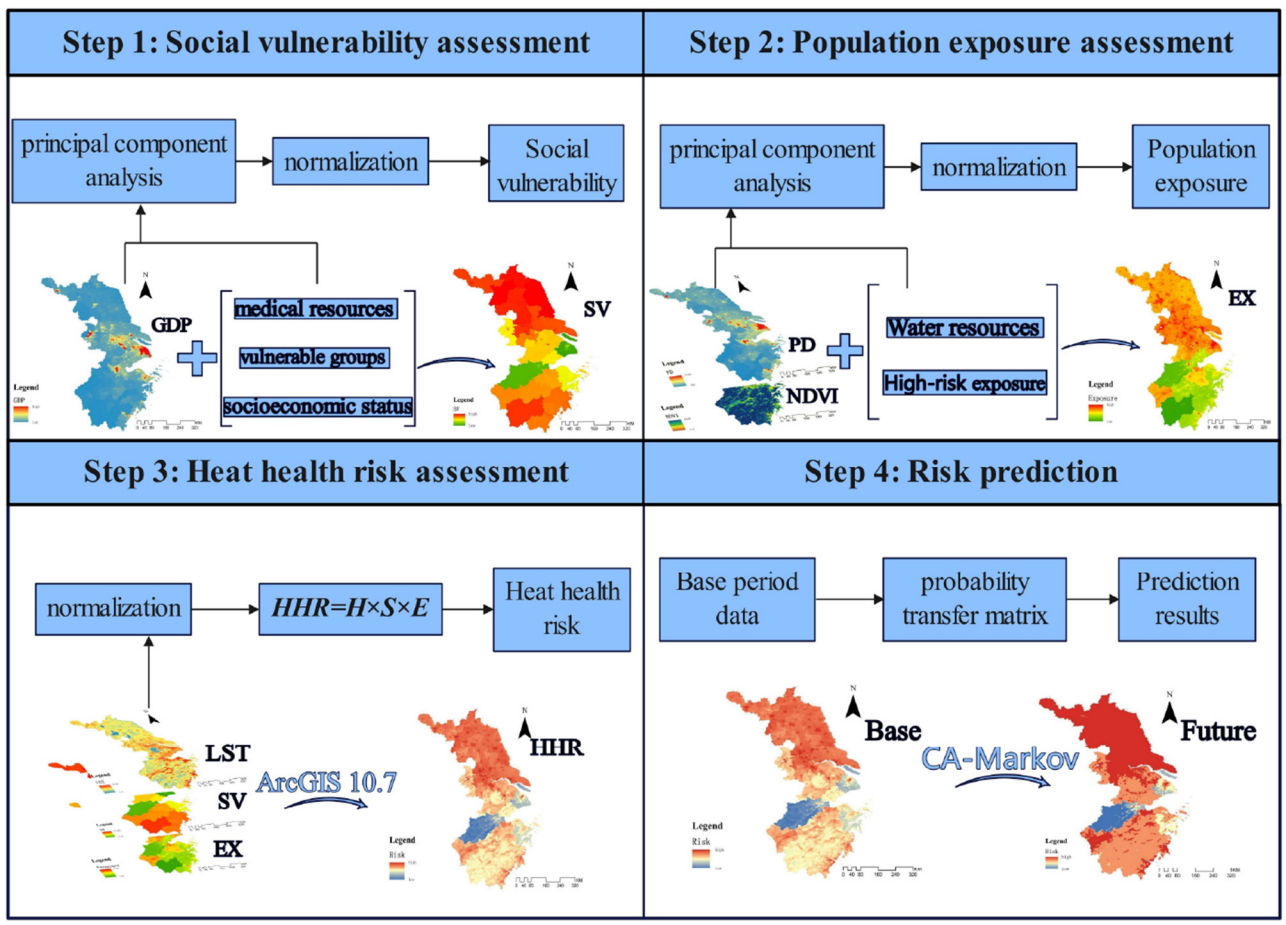
Study framework.

In this study, based on the IPCC AR6 and the risk triangle framework, 13 indicators of different types were selected for hazard, vulnerability and exposure (Table 1). At the hazard level of this study, nighttime LST was selected to reflect the heat hazard (38). In terms of vulnerability, this study selects physicians, hospital beds, older adults and female populations, and unemployment rate, which can represent vulnerable groups. GDP and disposable income accurately reflect level of socio-economic development. Besides, physicians and hospital beds were also considered to reflect the accessibility of health resources. At the exposure level, population density, agricultural and construction practitioners are selected to reflect the heat exposure intensity of human. NDVI and water resources can reflect the resistance level to exposure. Figure 2 illustrates the entire process of the study framework.

### 3.4 Statistical analyses and indicators

PCA integrates multiple indicators into a few principal components through dimensionality reduction, and determines the weight of each indicator based on the variance contribution rate of each principal component (20). In this study, the weights of each indicator are calculated based on the variance contribution rate and loading of each principal component. Finally, the weight values of each indicator are obtained through normalization processing. On this basis, the weight is calculated as follows:


$$\begin{array}{l} b_{ij}=\frac{a_{ij}}{\sqrt{X_{j}}}\\ W_{i}=\frac{b_{i1}PC_{1}+b_{i2}PC_{2}+b_{i3}PC_{3}\cdot \cdot \cdot +b_{ij}PC_{j}}{PC_{1}+PC_{2}+PC_{3}\cdot \cdot \cdot +PC_{j}} \end{array}$$

Where *W*_*i*_ is the weight of *i-*th indicator; *PC*_*j*_ is the variance contribution rate of *j*-th principal component; *a*_*ij*_ is the loading of the *i*-th indicator and the *j*-th principal component; *X*_*j*_ is the eigenvalue of the *j*-th principal component.

Accordingly, the risk of heat hazard is obtained by multiplying (39, 40) the three indicators of heat hazard, social vulnerability and exposure (41) as follows:


$$\begin{array}{l} HHR=H\times S\times E \end{array}$$

where *HHR* is a composite score of the risk to human health from *H*, and *S* and *E* are the results of the assessments of heat hazard, social vulnerability and exposure, respectively.

All the assessment indicators all require standardization, which is carried out using the following formula:


$$\begin{array}{l} X_{p}=\frac{X-MIN}{MAX-MIN}+0.01\\ X_{n}=\frac{MAX-X}{MAX-MIN}+0.01 \end{array}$$

where *X*_*p*_ is the normalized value of the positive indicator, *X*_*n*_ is the normalized value of the negative indicator in the range [0.01, 1.01], *X* is the original value, and *MIN* and *MAX* are the minimum and maximum values of the original value, respectively. The HHR is normalized to [0, 1].

### 3.5 HHR classification

In this study, the natural breakage classification (NBC) is used to classify HHR to distinguish different levels of health risk. NBC divides data into multiple categories in such a way that the variation within each category is minimized while the variation between categories is maximized. This method seeks the optimal grouping scheme for data by minimizing the within-class variance and maximizing the between-class variance. Based on the natural distribution characteristics of the data, it can reveal the inherent structure of the data, facilitating visualization and analysis. In this study, the HHR in 2010 is classified by natural breakage classification. As the classification standard (Table 2) of HHR in this paper, HHR is divided into five categories: lowest-risk area, low-risk area, medium-risk area, high-risk area and highest-risk area. After classification, ArcGIS is used to visualize the spatial distribution of HHR.

**Table 2:** Classification of the heat health risk.

**HHR**	**Lowest**	**Low**	**Medium**	**High**	**Highest**
Grading standard	<0.2	0.2–0.4	0.4–0.5	0.5–0.58	>0.58

### 3.6 Model prediction

In this study, the CA-Markov model is used for the simulation, prediction and analysis of heat health risk. CA-Markov model is a process theoretical model based on the Markov random process system so as to achieve the purpose of prediction and random control. CA consists of discrete cells of time, space and state that simulate spatiotemporal evolution through a certain transformation rule (42). The evolution of the CA simulation is as follows:


$$\begin{array}{l} S_{ij}^{t+1}=f_{ca}\left(S_{ij}^{t}\right) \end{array}$$

where $S_{ij}^{t}$ is the state of the *ij*-th cellular at moment *t*, *f*_*ca*_ is the cellular transformation function, and $S_{ij}^{t+1}$ is the state of the *ij*th cellular at moment *t* + 1.

The rationale of the Markov model is to use the probability of transfer between the initial and intermediate states to predict the trend at time *t* (43). The Markov forecasting process is as follows:


$$\begin{array}{l} S^{t+1}=S^{t}\times P\\ P=\left[\begin{array}{ccc} P_{11} & \cdots & P_{1j}\\ \vdots & \ddots & \vdots \\ P_{i1} & \cdots & P_{ij} \end{array}\right] \end{array}$$

where *S*^*t*^ is its state at moment *t*, *P* is the probability transfer matrix, and *S*^*t*+1^ is its state at moment *t*.

The CA-Markov model can take advantage of both the CA model's ability to simulate changes in spatial systems and the Markov model's ability to make long-term predictions; that is, the simulation of health risk prediction is realized by adding the spatial distribution elements with continuous properties to the analysis process of the Markov chain There are three steps in conducting the prediction: (1) The transfer probability matrix and distribution conditional probability images were obtained from the two-period health risk data analysis. (2) The CA filter is constructed to create weighting factors based on the central cell and surrounding neighboring cells, thereby determining the rule of variation for the central cell state. (3) The prediction base period is selected, and prediction based on the health risk probability transfer matrix in step (1) is conducted. This study uses the CA-Markov module in IDRISI and ArcGIS to complete the process of CA-Markov prediction.

## 4 Results

### 4.1 Principal component analysis

As shown in Table 3, we conducted tests to determine whether the data were suitable for factor analysis. According to the results, the KMO value is >0.7 and *P* < 0.05, indicating a significant test result. This suggests that the data are suitable for factor analysis and that PCA can proceed.

**Table 3:** KMO and Bartlett's test.

**KMO measure of sampling adequacy**		**0.702**
Bartlett's test of sphericity	Approx Chi-square	728.619
	Df	66
	Sig.	0.000

The PCA results are shown in Table 4. After conducting the PCA with targeted rotation in each year, four meaningful components were derived, explaining over 80% of the total variance of selected indicators.

**Table 4:** Results of the PCA of social vulnerability and exposure.

**PC**	**Eigenvalues**	**Contribution ratio of variance**	**Cumulative contribution of variance**
1	5.126	42.714%	42.714%
2	1.849	15.405%	58.119%
3	1.691	14.094%	72.212%
4	1.163	9.692%	81.905%

After calculating the variance contribution rates and loadings of each principal component, normalization processing was finally conducted to obtain the weight values for each indicator. The results are shown in Table 5.

**Table 5:** Weights of the PCA of social vulnerability and exposure.

**Factor category**	**Indicator**	**Weight**
Social vulnerability	GDP (SV1)	0.14
	Disposable income (SV2)	0.17
	Physicians (SV3)	0.20
	Hospital beds (SV4)	0.16
	Older adults (SV5)	0.13
	Female (SV6)	0.11
	Unemployment (SV7)	0.09
Human exposure	NDVI (EX1)	0.12
	Population density (EX2)	0.32
	Water resource (EX3)	0.30
	Agricultural practitioners (EX4)	0.01
	Construction practitioners (EX5)	0.25

### 4.2 Heat health risk

**Figure 3:**
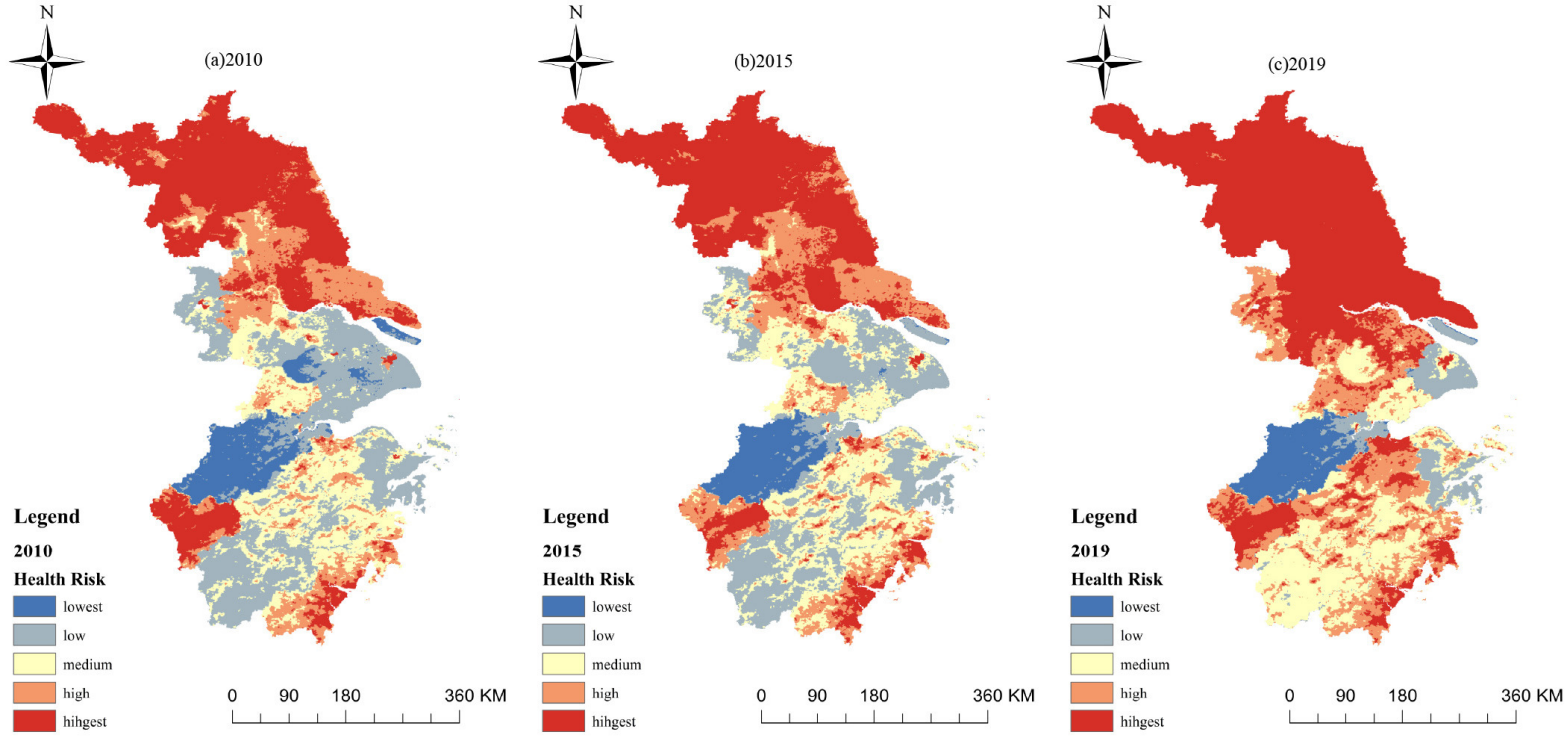
Spatial distribution of HHR in the eastern China. **(A)** 2010. **(B)** 2015. **(C)** 2019.

The results of this study show that the heat health risk in most areas of eastern China is mainly highest risk (Figure 3). In 2010, 2015 and 2019, the proportion (Table 6) of highest risk areas and medium risk areas reached 50.16%, 53.11% and 67.48%, respectively, with a stable upward trend. The area proportion lowest risk areas has decreased by 0.95% in the decade 2010–2019, the area of high risk areas has increased by 2.67% in the 9 years, and the area of low risk areas has changed most significantly, reducing by 19.06%.

**Table 6:** The proportion of HHR.

**Risk level**	**2010**	**2015**	**2019**
Lowest risk	7.57%	6.49%	6.62%
Low risk	25.24%	21.39%	6.18%
Medium risk	19.05%	21.53%	19.04%
High risk	17.04%	19.01%	19.71%
Highest risk	31.11%	31.58%	48.44%

According to the spatial distribution pattern of the heat health risk, the area with highest risk in eastern China accounts for a relatively large proportion (Figure 3). In terms of overall hierarchical distribution, the areas with high risk are mainly distributed in the southeast and southwest of Zhejiang, the north of Jiangsu and the central urban area of the city. The areas with low risk are concentrated in the southern mountainous area, the suburbs and rural areas and the water source area, which generally shows that the risk in the north and southeast are high and the risk in the east and central-west are low. From the perspective of time scale, the risk level of the whole north of Jiangsu Province was high risk from 2010 to 2019, and the area of high-risk areas shows increasing trend. The risk level of large and medium-sized urban areas has been high over the 9 years due to the significant impact of urbanization. The areas with low risk in the northwest of Zhejiang have maintained a relatively low level of risk over the past 9 years, and the area of overall lowest and low risk areas has decreasing trend over time.

**Figure 4:**
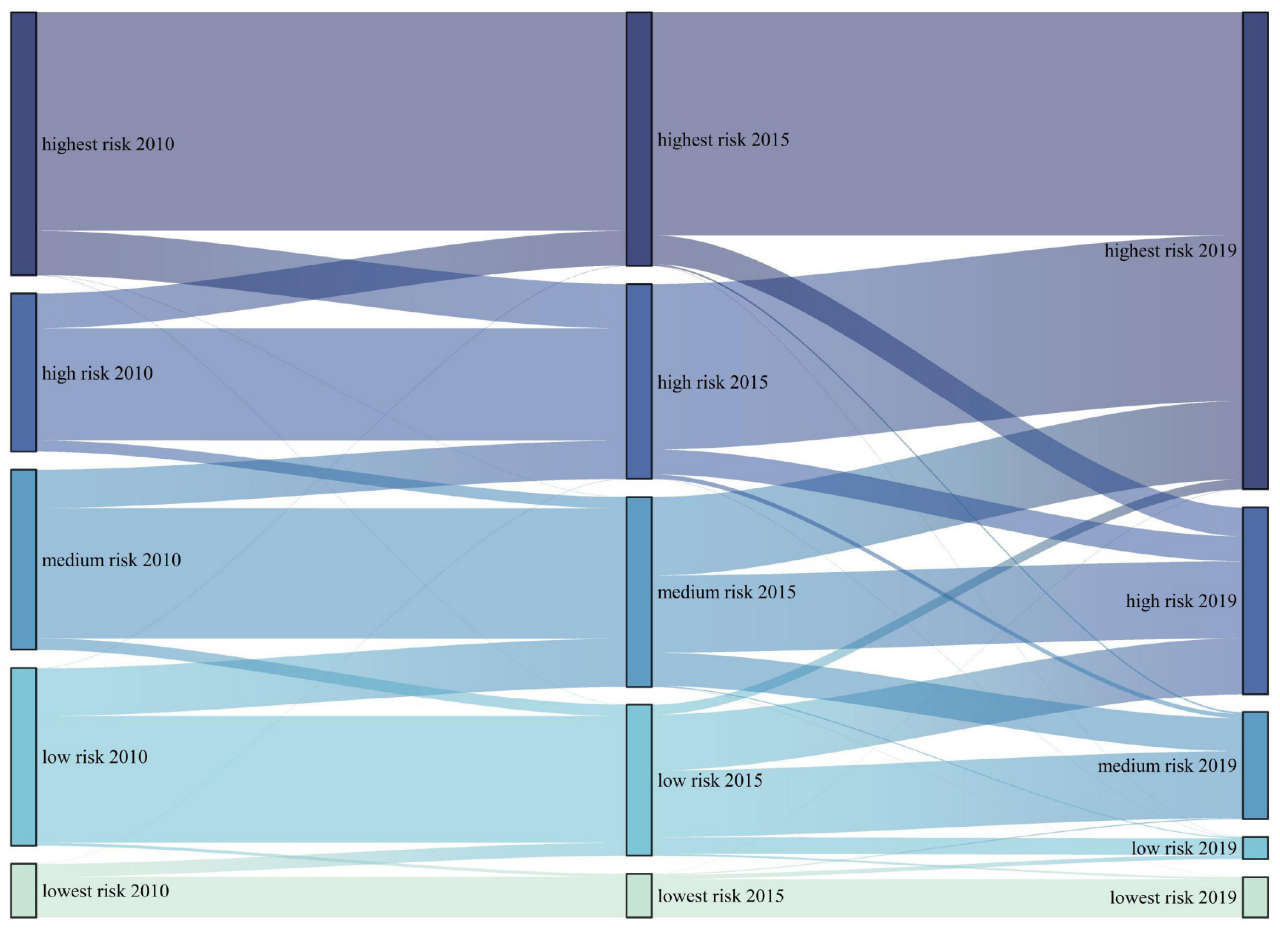
Area conversion of HHR in the eastern China.

By calculating the area transfer between 2010, 2015 and 2019, it can be found that the area conversion of different heat health risk areas mainly occurs between adjacent levels (Figure 4). For example, the low-risk areas in 2015 mainly come from the low-risk areas, lowest risk areas and medium risk areas in 2010; most of the lowest-risk areas in 2019 come from the original lowest-risk areas and low-risk areas. It also shows that there is usually no mutation process in the transformation of risk. The types of scale transformation mainly include the transformation of low and medium risk areas, the transformation of medium and high-risk areas, and the transformation between high and highest risk areas.

**Figure 5:**
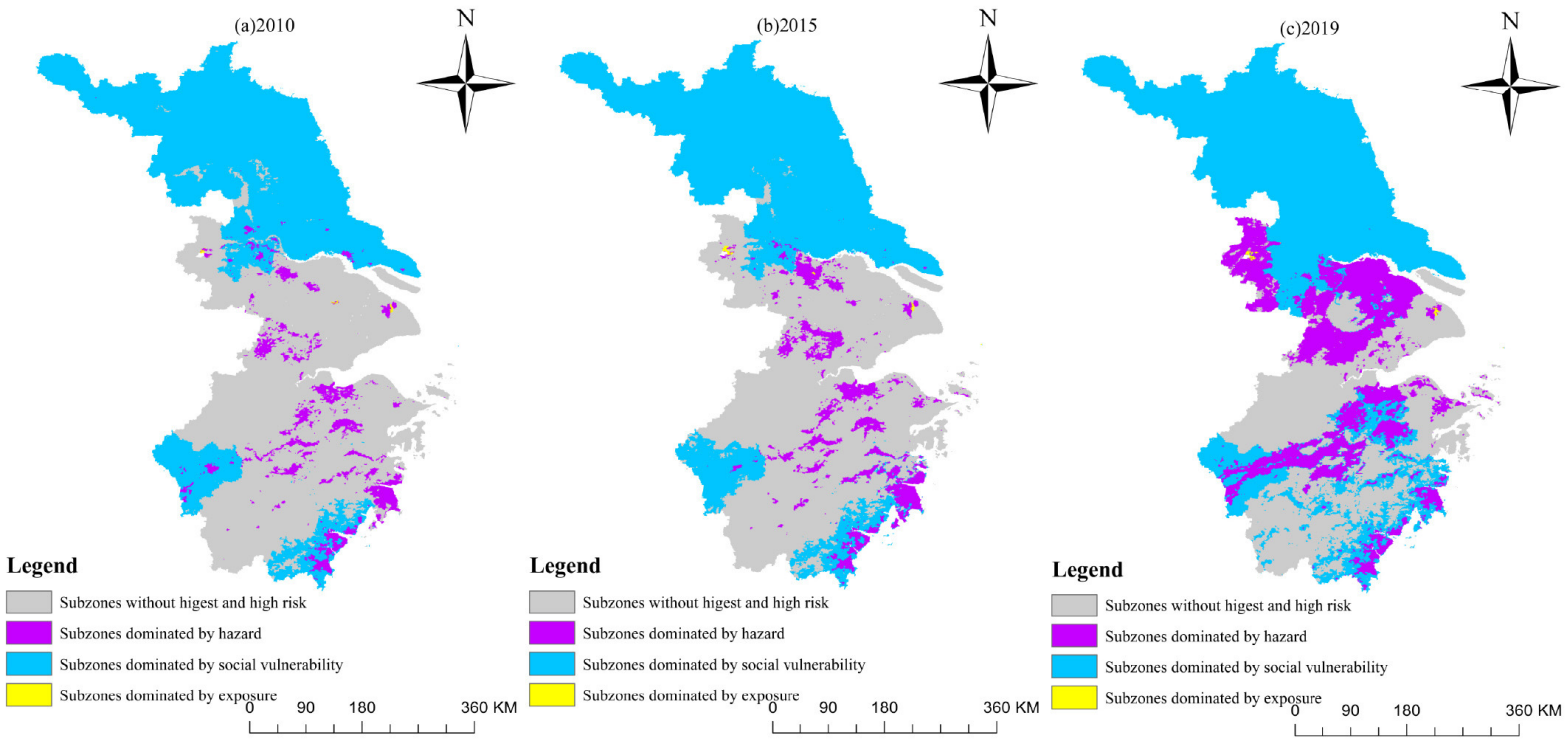
Spatial distribution of HHR subzones in the eastern China. **(A)** 2010. **(B)** 2015. **(C)** 2019.

Furthermore, the map in Figure 5 illustrates the distribution of highest-risk and high-risk areas based on the leading indicators among hazard, social vulnerability, and exposure. It emphasizes that the areas dominated by social vulnerability are the largest, mainly located in western Jiangsu Province and the southeastern coastal areas of Zhejiang Province. The hazard-dominated areas are mainly distributed around Taihu Lake, extending northeast to southwest of Zhejiang, as well as along a coastal stretch in southeast Zhejiang. In contrast, the exposure-dominated areas are concentrated mainly in the central regions of Shanghai and Nanjing. Table 7 shows hazard-dominated areas increased by 15.98%, social vulnerability-dominated areas decreased by 16.00%, and exposure-dominated areas stayed stable around 0.10%. This confirms that HHR is primarily influenced by the distribution of hazards and social vulnerability.

**Table 7:** The proportion of areas with the highest and high-health risk dominated by each category.

**Category**	**2010**	**2015**	**2019**
Hazard	11.42%	13.92%	27.40%
Social vulnerability	88.49%	85.93%	72.49%
Exposure	0.09%	0.15%	0.11%

### 4.3 Risk prediction

**Figure 6:**
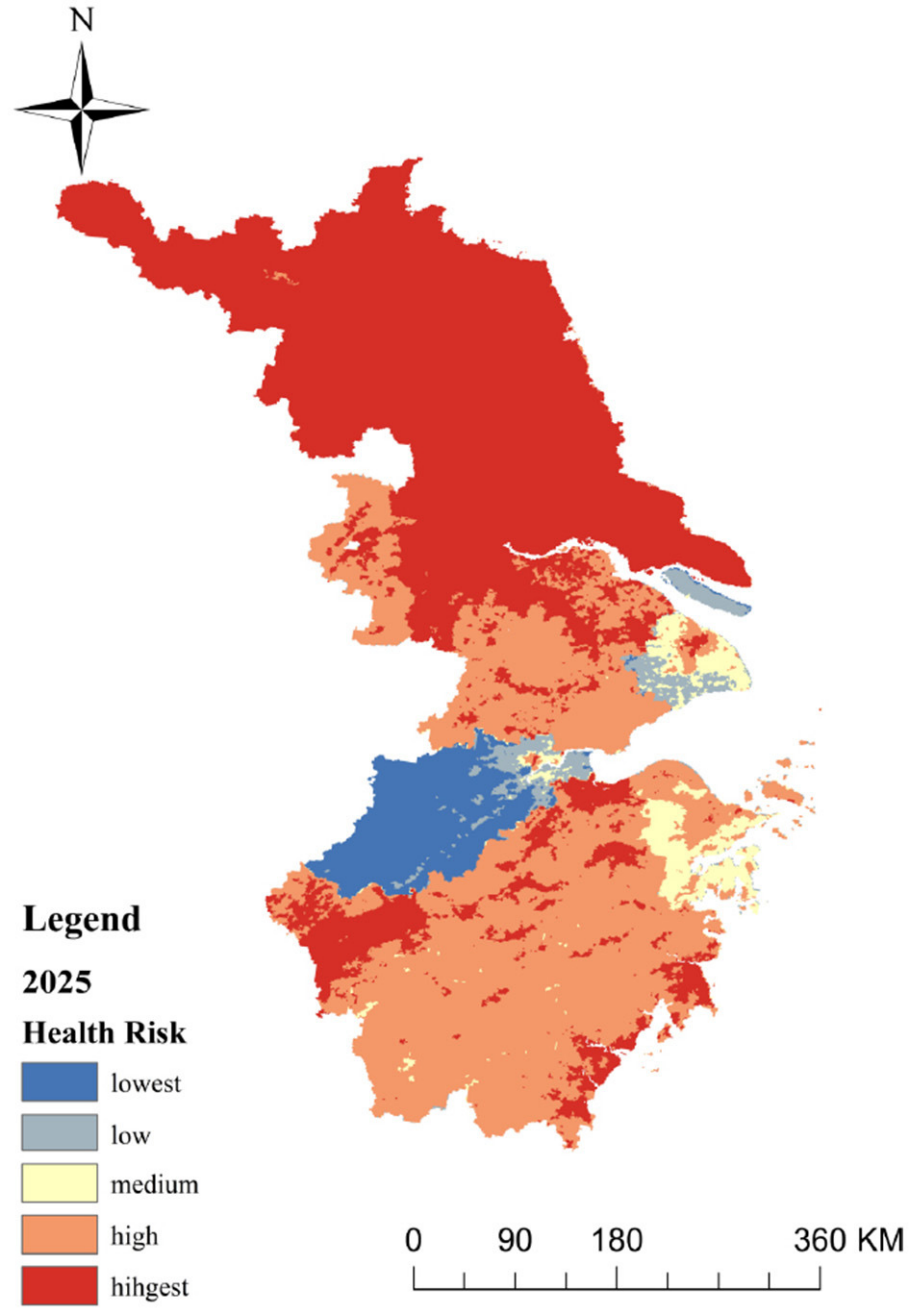
Spatial distribution of social vulnerability in the eastern China.

Using the CA-Markov module in IDRISI, based on the heat health risk results in 2010, 2015 and 2019, taking 2010 as the starting year, we calculated the heat health risk level area transfer matrix from 2010 to 2015, predicted the heat health risk of eastern China in 2019, compared it with the actual data, and calculated the Kappa coefficient of 0.882 with *P* < 0.01, indicating that the prediction results are highly consistent with the actual situation (Table 8). We took 2015 as the starting year, where based on the heat health risk area transfer matrix from 2015 to 2019 and the generated suitability Atlas of various risk levels, we assumed that the heat health risk level transfer trend from 2010 to 2019 is basically similar to that 9 of years later, and a 5 × 5 mole neighborhood is used as the filtering parameter of the CA-Markov model. The operation of the model is an iterative cycle every year. Through 10 iterations, the spatial data set of heat health risk in eastern China in 2025 is simulated, and the spatial distribution prediction map is formed (Figure 6).

**Table 8:** The result of consistency test.

**Kappa coefficient**	***P***
0.882	0.000

This paper tests the robustness of CA-Markov the model by varying the number of cellular automaton iterations. The test results are presented in Table 9. Whether increasing or decreasing the number of iterations, the Kappa coefficients are similar to the result in Table 8, verifying the robustness of the CA-Markov model.

**Table 9:** The results of robustness test.

**Number of iterations**	**Kappa coefficient**	***P***
8	0.879	0.000
12	0.881	0.000

The prediction results (Figure 6) show that the distribution pattern of risk level in eastern China in 2025 is consistent with that of the past 9 years. The statistics show that the proportions of five levels of health risk areas in 2025 are as follows: lowest-risk areas are 6.64%, low-risk areas are 2.46%, medium risk areas are 3.67% and high-risk areas are 38.78%. While the area of highest-risk areas is accounting for 48.45% of the total area, this level is far higher than the data of 9 years ago. The overall distribution pattern is high-risk areas, mainly expanding outwards from cities, creating larger high-risk areas. Meanwhile, low-risk areas of Shanghai are shifting to medium-risk, and medium-risk areas in northern Zhejiang are growing.

## 5 Discussion

**Figure 7:**
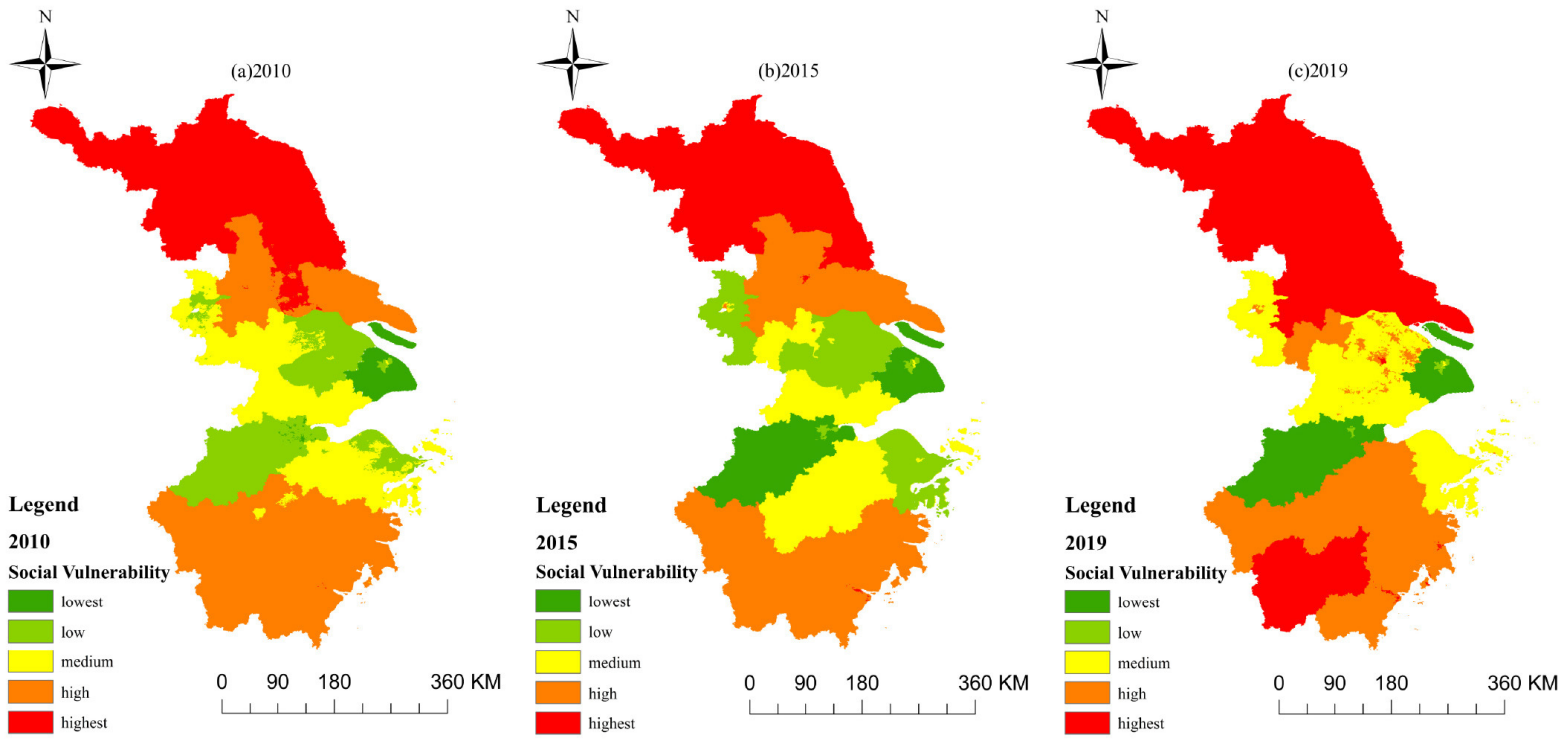
The prediction of HHR in the eastern China. **(A)** 2010. **(B)** 2015. **(C)** 2019.

Areas with high levels of social vulnerability (Figure 7) not only lack health resources and suffer from economic underdevelopment, but are also affected by other factors (44). These factors may include income inequality, education levels, access to social services, and environmental resilience, among others. Conversely, regions with low vulnerability tend to boast robust economic growth and ample health resources, reflecting a more equitable and developed societal structure. For instance, Nantong's increasing social vulnerability can be attributed not only to population aging but also to factors such as limited social welfare programs and inadequate healthcare infrastructure. Medium vulnerability areas present an intriguing paradox: they may enjoy high economic development and low unemployment rates, yet lag behind in health resources (45). Rapid urbanization (46), often accompanied by environmental degradation and social disparities, exacerbates this vulnerability. The influx of migrants, without adequate support systems, can strain public services and exacerbate social tensions, further increasing the area's overall vulnerability. Regions characterized by low social vulnerability, such as the Yangtze River Delta, typically exhibit not only high levels of economic development but also a well-rounded approach to societal wellbeing (47). They invest heavily in education, healthcare, and environmental sustainability, creating a resilient social fabric that can withstand external shocks. These areas also tend to have more equitable income distributions and robust social safety nets, which further mitigate vulnerability.

High-risk areas, despite their advantageous locations, abundant health resources, and economic development, face heightened heat health risks and exposure due to a complex interplay of factors (48). Their dense land use patterns contribute to higher temperatures, exacerbated by the significant heat storage capacity of urban structures (49). Additionally, the large construction workforce, often working outdoors in harsh conditions, further amplifies their vulnerability. The interaction between urbanization, industrialization, and socio-economic status creates a multifaceted landscape where heat hazards are driven not just by physical characteristics but also by societal structures and behaviors (50).

Conversely, low-risk areas, which tend to be less economically developed and more reliant on agriculture, animal husbandry, and fishing, exhibit a different set of dynamics. While they lack the health resources available in urban centers, they benefit from lower population densities, which reduce the concentration of heat-generating activities. Their greater green cover and abundant water resources provide natural cooling mechanisms (51), mitigating heat hazards and exposure (52). However, it is important that these benefits are not absolute, and low-risk areas can still experience heatwaves and related health impacts, albeit to a lesser extent. The interplay between natural environmental factors and human activities, such as land-use changes and deforestation, can introduce uncertainties and variations in heat health risks even within these seemingly lower-risk regions.

Some Chinese researchers primarily conduct their studies through questionnaires and statistical analysis, focusing on residents' perceptions of heatwave risks in Guangdong, adaptive behaviors, and experiences with heatstroke (53). In contrast, some researchers concentrate on exposed areas and population dynamics in Beijing (54), employing a variety of methods such as questionnaires, remote sensing analysis, and morphological spatial pattern analysis (55). They also specifically introduce dynamic population data and the exposure-response relationship between temperature and health to delve deeper into the identification of heat-exposed areas, heat risk assessment, and the impact of population dynamics on heat exposure. Ma et al. have expanded their research to 66 communities in China, emphasizing the impact of heatwaves on mortality and the modulating effects of individual and community characteristics (56).

In terms of theory, the study provides a comprehensive examination of the spatial and temporal distribution patterns of heat health risk. On one hand, by identifying key areas of highest and high risk, as well as those with low and lowest risk, the research contributes to the understanding of how environmental, social, and urbanization factors interplay to influence health risks associated with heat. On the other hand, the study's focus on the transformation of risk areas over time adds a dynamic perspective to the theoretical discussion. By showing that area conversions mainly occur between adjacent risk levels, the research contributes to the understanding of how risk can evolve and change, highlighting the need for adaptive and responsive risk management strategies.

In terms of practice, the study's findings offer valuable insights for policymakers. On one hand, by identifying high-risk areas, the research provides a basis for targeted interventions aimed at mitigating the impacts of heat on human health. This includes measures such as improving urban green spaces, enhancing public awareness about heat-related health risks, and developing early warning systems. The identification of different factors-dominated areas suggests that policies should also focus on reducing the vulnerability of certain populations to heat risks. On the other hand, the study's prediction results for 2025 offer a forward-looking perspective that can inform long-term planning and strategy development. By highlighting the expansion of high-risk areas and the shifting of risk levels in specific regions, the research provides a roadmap for proactive risk management and mitigation efforts.

### 5.1 Policy suggestions

To significantly reduce health risks associated with urban environments, a comprehensive and targeted approach is essential. In central cities, urban planning interventions should focus on alleviating the heat island effect by planting more trees and shrubs in public spaces and along streets (57), expanding urban parks and green spaces, and creating cooler microclimates through the expansion of water bodies such as fountains, lakes, and rivers (50, 52). Additionally, green building designs that incorporate natural lighting (58), ventilation, and energy-efficient materials should be promoted, along with the installation of green roofs and walls to provide insulation and reduce heat absorption.

Relocating industrial factories to the outskirts of the city can further minimize greenhouse gas emissions (59) and enhance the urban environment (60), while robust transportation networks and employment opportunities in the suburbs can encourage population decentralization and reduce urban density (18, 61). Social support plans should include policies and allowances for outdoor workers to avoid working during peak heat hours, ensuring access to shaded rest areas and cool drinking water (62). Workers in agriculture and forestry should be provided with necessary cooling equipment and protective gear, along with regular health check-ups and training sessions on heat stress management.

For older adults and vulnerable populations (16), specialized facilities should be constructed to ensure access to air-conditioned spaces and medical care, with financial subsidies offered for the installation of cooling equipment in care institutions (63). Public health activities should focus on improving medical facilities in underserved areas through subsidies, staff transfers, training programs, and the importation of skilled technicians (64, 65). Comprehensive public awareness campaigns should be launched in less developed regions to educate the population on heat risks and preventive measures, utilizing local media, community centers, and schools to disseminate information and promote healthy behaviors.

Economic incentives and support should be provided to less developed regions to foster economic growth and resilience against heatwaves, ensuring that policy frameworks include measures to mitigate and adapt to rising temperatures. In suburban areas, zoning regulations should be implemented to prevent excessive land development that could lead to the loss of green spaces and water bodies (66, 67), while encouraging the preservation of natural landscapes and the creation of green corridors to mitigate heat. By taking these specific and targeted steps, urban and suburban areas can enhance the overall quality of life for residents while significantly reducing health risks associated with heat and other urban hazards.

### 5.2 Limitations

There are several limitations and uncertainties in the study. Firstly, the selection of indicators involves various factors, including meteorological, economic, demographic, and social factors. However, some indicators were not chosen due to limitations in the data sources and spatial representation. Additionally, the selection of indicators might have influenced the assessment results. There is no standardized approach for selecting indicators. Understanding heat health risks requires a nuanced approach that accounts for the intricate interactions between physical, socio-economic, and environmental factors. Simplistic explanations fall short of capturing the full complexity of these issues. Therefore, to conduct a deeper analysis of social vulnerability, it is imperative to employ a more nuanced vulnerability index that incorporates a multitude of interconnected factors, including not just economic indicators but also social cohesion, environmental health, and governance effectiveness. Future research should refine selection methods for robustness, representativeness, and applicability, incorporating such indices. Methods such as the Heat Vulnerability Index (33), the Hesitant Analytic Hierarchy Process (34), and LST-based models (68) offer diverse perspectives but require standardization and integration to form a comprehensive assessment framework. The CA-Markov model, while effective in simulating and predicting spatial-temporal changes, exhibits several limitations. Its predictions are inherently uncertain due to assumptions about the stationarity of transition probabilities and the potential oversimplification of complex system dynamics. Additionally, the model's reliance on historical data can lead to inaccuracies if the data is incomplete or does not accurately represent the system's true behavior. Furthermore, incorporating heat-related death statistics enhances risk area validation. Further research is crucial to improve accuracy and precision, including exploring advanced modeling techniques, enhancing data resolution, and refining our understanding of the multifaceted nature of heat health risks.

## 6 Conclusion

In conclusion, our research introduces an innovative method for assessing urban heat-related health risks, which effectively facilitates risk zoning and prediction. Our findings reveal that risks decline from urban to rural areas, suggesting that urban centers require balanced planning strategies to mitigate heat islands, such as promoting green infrastructure initiatives like increased tree coverage and green roofs. In contrast, suburbs necessitate the development of targeted social support plans for vulnerable populations.

This series of findings unveils the specific spatial characteristics of heat health risks in urban environments, demonstrating that these risks are associated not only with the density and temperature of urban centers, but also influenced by various factors such as vegetation cover and economic development levels. These discoveries offer deeper insights into the formation mechanisms of urban heat island effects and the vulnerability of different socioeconomic groups to heat health risks. Consequently, our research not only provides spatial distribution maps of these risks but also introduces a new perspective for understanding the complexity and diversity of urban heat health risks. Firstly, they serve as a basis for optimizing monitoring systems, enabling them to more accurately identify and issue early warnings for high-risk areas. Secondly, by integrating multiple data sources and risk assessment methods, our study paves the way for the intelligence and automation of warning systems. Moreover, these findings reveal distinct challenges faced by urban centers and suburbs during heatwaves, providing a foundation for developing targeted mitigation measures. For instance, in urban centers, measures like increasing greenery, improving building designs, and urban planning may be necessary to reduce heat island effects; whereas in suburbs, additional social support and shelters for vulnerable groups could be required. Furthermore, these discoveries underscore the importance of community engagement and public education in raising awareness and enhancing residents' capacity to cope with health risks. Lastly, our research provides scientific evidence for policymakers to formulate more comprehensive and effective heatwave mitigation plans.

However, it is important to acknowledge the limitations of our study, including data availability and quality constraints, methodological assumptions, and the potential for unmeasured confounding factors. In the future, we will further refine our research.

Firstly, the impact of climate change on future heat health risks should be investigated to anticipate and prepare for evolving threats. Secondly, more sophisticated vulnerability assessments that incorporate a broader range of social and demographic factors. Lastly, by evaluating the effectiveness of different heatwave mitigation strategies, we can provide information for policymakers and improve public health outcomes. By addressing these areas, we can further refine our responses to heat-related health risks, ultimately contributing to improved public health in urban and suburban environments.

## Data Availability

The original contributions presented in the study are included in the article/supplementary material, further inquiries can be directed to the corresponding author.
